# A Case of Concurrent Leukocytosis and Systemic Capillary Leak Syndrome Due to Pegfilgrastim

**DOI:** 10.7759/cureus.24640

**Published:** 2022-05-01

**Authors:** Nico Gotera, Shayee Hasan, Pritee Shrestha, Caio Heleno, Amy Tesar

**Affiliations:** 1 Internal Medicine, MercyOne North Iowa Medical Center, Mason City, USA

**Keywords:** neulasta, leukocytosis, pegfilgrastim, neupogen, filgrastim, granulocyte colony-stimulating factor, systemic capillary leak syndrome

## Abstract

Pegfilgrastim is a granulocyte colony-stimulating factor agent used in patients receiving myelosuppressive therapy with chemotherapy or radiation. Two adverse effects associated with this agent include capillary leak syndrome and leukocytosis. To our knowledge, this is the first case of a patient who developed both systemic capillary leak syndrome and leukocytosis greater than 100,000 cells/µL after receiving pegfilgrastim. This patient received early fluid resuscitation, vasopressor support, and methylprednisolone, which improved her clinical course during hospitalization.

## Introduction

Pegfilgrastim is a granulocyte colony-stimulating factor (G-CSF) that works by stimulating the production and maturation of neutrophil progenitors while also promoting their release into the bloodstream. It is considered to be a long-acting form of filgrastim, another G-CSF agent, through covalent bonding of polyethylene glycol, which increases its duration of action. Indications for these agents include patients at risk of developing febrile neutropenia while receiving myelosuppressive chemotherapy or radiation [1]. Two adverse effects described for these agents are systemic capillary leak syndrome and leukocytosis [2]. Systemic capillary leak syndrome is a syndrome associated with hypotension, albuminemia, and hemoconcentration due to extravascular leakage [3]. Leukocytosis is defined as white blood cells (WBCs) greater than 11,000 cells/µL [4]. We present a case of a 58-year-old female who developed concurrent systemic capillary leak syndrome and leukocytosis greater than 100,000 cells/µL after receiving pegfilgrastim.

## Case presentation

A 58-year-old female with a past medical history of limited-stage small cell lung cancer of the right upper lobe was directly admitted to the critical care unit from an outside facility after a presyncope fall and persistent hypotension. She was originally diagnosed with small cell lung cancer through a right lung biopsy three months prior to presentation. After completing staging computed tomography (CT) of the chest, abdomen, and pelvis, magnetic resonance imaging (MRI) of the brain, and positron emission tomography (PET)/computed tomography scan, she was staged as group IA2 and limited-stage small cell lung cancer. Her systemic chemotherapy involved cisplatin, etoposide, dexamethasone, and pegfilgrastim for a total of four cycles. This regimen was chosen based on the National Comprehensive Cancer Network guidelines for small cell lung cancer. Pegfilgrastim was administered due to the side effect of myelosuppression on this regimen [5]. She was also treated with radiation therapy over one week with intensity-modulated radiation therapy/stereotactic body radiation therapy to a dose of 60 Gray Units (Gy) in five fractions with 6x photons. She completed and tolerated radiation well without any toxic effects. She started systemic chemotherapy the day after the completion of radiation.

The patient started cycle three of systemic chemotherapy five days prior to admission. Notably, she received pegfilgrastim 6 mg/0.6mL on the day before admission. She did not tolerate the previous dose of pegfilgrastim well due to the development of bone pain, which is a common side effect of pegfilgrastim and filgrastim [1,2]. On the day of admission, she presented to an outside facility emergency department with lightheadedness after standing up and fell without any loss of consciousness or trauma to her head. This was associated with headache, malaise, cough with sputum, right knee pain, and new-onset urinary retention. Her shortness of breath worsened after starting systemic chemotherapy but had not changed since then. Her blood pressures at the outside facility were notably hypotensive, with systolic blood pressures remaining around 70s on three separate checks despite receiving 3L of fluids. She received meropenem and ceftriaxone at the outside facility emergency department before being transferred to the critical care unit at our facility that same day for a higher level of care.

The patient’s past medical history was also notable for paroxysmal atrial fibrillation, heart failure with a preserved ejection fraction of 55-60%, chronic obstructive pulmonary disease (COPD) on 1 L/min of oxygen at home, pulmonary hypertension, asthma, and active smoking history. Her home medications included digoxin, albuterol, ipratropium, aspirin, apixaban, carvedilol, gabapentin, nortriptyline, pantoprazole, quetiapine, topiramate, torsemide, tramadol, trazodone, and bupropion. The patient's physical exam was remarkable for a pulmonary exam of diminished breath sounds bilaterally with end-expiratory wheezing. There were no other breath sounds, tachypnea, or accessory muscle use noted. The patient also had 2+ pitting edema of the bilateral lower extremities but no evidence of jugular venous distention. Her cardiovascular exam was noted to be in regular rate and rhythm. She had normal S1 and S2, without murmurs, gallops, or rubs.

Her initial labs on admission were notable for WBCs 115,400 cells/µL with neutrophil percent of 90.2% and lymphocyte percent of 3.6%, hematocrit 29.8%, albumin 3.0 g/mL with unremarkable electrolytes, cortisol, troponin, thyroid function, lactate, procalcitonin, brain natriuretic peptide, and digoxin levels. Urinalysis, respiratory viral panel, sputum culture, urine culture, and blood cultures did not show any signs remarkable for infection. Electrocardiograms did not show any arrhythmias. CT of the head without contrast did not show any acute intracranial changes. CT of the chest, abdomen, and pelvis with contrast showed posterior left lung base atelectasis, trace chronic pleural fluid, emphysema with suspected pulmonary hypertension, and no evidence of pulmonary embolism or focal consolidations concerning for pneumonia. Transthoracic echocardiogram showed an ejection fraction of 65-70% with abnormal relaxation diastolic filling pattern with difficulty in ascertaining right ventricular systolic pressure.

**Figure 1:**
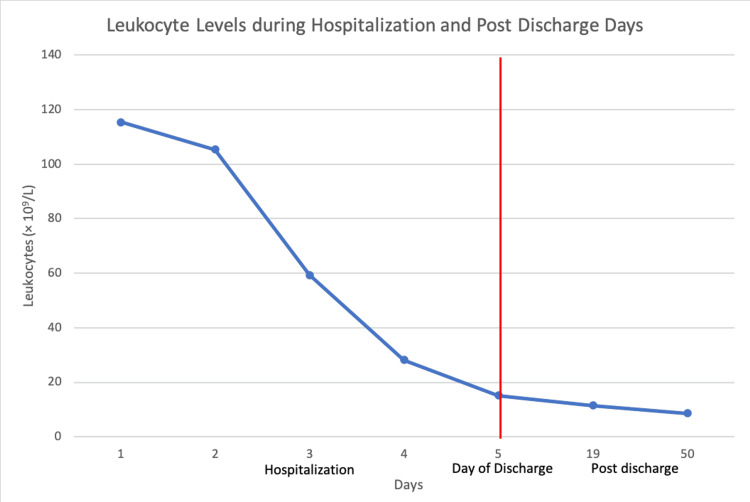
Leukocyte levels during hospitalization and post discharge days Red line represents the day of discharge

Given the complex history and presentation of the patient, she was started on maintenance fluids, vasopressor support with a low dose of norepinephrine, and broad-spectrum antibiotics, including vancomycin and piperacillin-tazobactam for presumed septic shock. She was also started on scheduled albuterol and ipratropium, intravenous methylprednisolone, and azithromycin out of concern for COPD exacerbation. During her hospitalization, her WBC count dramatically improved. She was tapered off vasopressor support on day two, and maintenance fluids were discontinued on day three. Her antibiotics, except for azithromycin, were discontinued on day three. She was discharged four days later on oral prednisone and azithromycin for five days to treat her COPD. Her WBCs were notably 15,160 cells/µL on the day of discharge. She continued her chemotherapy with cisplatin, dexamethasone, and etoposide on discharge without pegfilgrastim and has been following up with oncology without any new concerns. Table 1 and Figure 1 detail remarkable labs and vital signs during hospitalization and after discharge.

**Table 1:** The change in blood pressure, leukocytes, hematocrit, and albumin during hospitalization and post discharge Norepinephrine of 1-2 mcg/L was started on day one and remained at this level until discontinued on day two. Maintenance fluids of lactate ringers was started on day one and discontinued on day three. Reference values are based on our laboratory's normal range.

Clinical sign or lab (reference values)	Hospitalization days					Days from discharge	
	1	2	3	4	5	19	50
Blood pressure (systolic mmHg/diastolic mmHg) (90-140 mmHg/65-90 mmHg)	73/48	97/62	149/78	133/78	138/73	94/64	102/67
Leukocytes (white blood cells x 10^3^/µL) (4.5-11.0 cells 10^3^/µL)	115.4	105.35	59.36	28.20	15.16	11.50	8.68
Hematocrit (%) (35-47%)	29.8	26.3	25.6	25.4	25.8	30.6	33.4
Albumin (g/dL) (3.5-5.0 g/dL)	3.0	2.5	2.6	3.7	3.0	3.8	3.2

## Discussion

G-CSF agents are often used in patients receiving myelosuppressive therapy with chemotherapy or radiation. However, there is limited data on pegfilgrastim or other G-CSF agents in causing adverse effects, such as systemic capillary leak syndrome and leukocytosis [1,2]. One systematic review involving capillary leak syndrome in sixty-two patients described 14.6% of those patients had G-CSF as the etiology for systemic capillary leak syndrome [3]. That same systematic review shared that leukocytosis, defined in the review as >15,000 WBCs/µL, occurred in eleven patients out of sixty-two patients. This systematic review does not describe the degree of leukocytosis. In another systematic review with 161 patients that experienced systemic capillary leak syndrome, there were 61 patients who developed leukocytosis, defined as >12,000 WBCs/µL in the paper [6]. Similarly, this systematic review does not describe the degree of leukocytosis for these patients. Alternatively, the manufacturers of filgrastim noted that there was an approximately 2% association of this agent with leukocytosis over 100,000 cells/µL [7].

Systemic capillary leak syndrome was first described by Clarkson et al. in 1960 and is typically described as reversible plasma extravasation and vascular collapse that is associated with a triad of hypoalbuminemia, hemoconcentration, and hypotension [3]. Other causes of shock are typically ruled out. More than two hundred and fifty cases have been described in the literature describing varying causes for systemic capillary leak syndrome, including idiopathic, drug-induced, malignancy, and other etiologies [8].

Based on the two separate systematic reviews, the most common presentations included hypotension (32.2-81.4%), edema (64.6-67.7%), and previous flu-like illness (34.2%) [3,6]. Typically, systemic capillary leak syndrome occurs in three phases: prodromal, fluid extravasation phase, and fluid recruitment phase [8]. Our patient had worsening malaise and flu-like illness consistent with the prodromal phase. Our patient had hypotension and edema consistent with the fluid extravasation phase. In addition, our patient had symptoms including dyspnea (11.2-27.4%) and syncope/pre-syncope (13.7%) [3,6]. Finally, the recovery phase can be associated with pulmonary edema and volume overload. Our patient did not develop either one of these complications. Of note, patients can also develop severe complications of acute renal failure, compartment syndrome, and rhabdomyolysis [8]. Our patient’s complex past medical history with heart failure and COPD made a diagnosis of systemic capillary leak syndrome more challenging due to overlap in symptoms with peripheral edema and dyspnea. There could have been a component of COPD exacerbation given her cough and sputum production, but this would not explain her symptoms of hypotension that were not responsive to fluids. In addition, there may be a possibility that the prodromal phase of systemic capillary leak syndrome could have triggered a potential COPD exacerbation. Her echocardiogram did not show any signs of reduced ejection fraction or other signs suggestive of cardiogenic shock. In regards to the new-onset urinary retention, the etiology of this is unclear, but neither systematic review mentions the development of urinary retention as a symptom of systematic capillary leak syndrome [3,6]. Her urinary retention resolved by the time the patient was discharged. The patient did have right knee pain upon her admission, but this was most likely due to her presyncope fall. In addition, it is worth noting that she did not have any mention of bone pain on follow-up with oncology one month and two months after discharge. The infectious workup was unremarkable and did not suggest septic shock or infection as a secondary cause of systemic capillary leak syndrome.

Regarding associated lab values, our patient had leukocytosis (40.1-87.1%) and hypoalbuminemia (84.4-96.9%), consistent with findings most commonly found in patients in the systematic reviews [3,6]. However, our patient lacked signs of hemoconcentration with decreased hematocrit. The lack of hemoconcentration was noted in a few patients in the systematic reviews (1-18.2%). For other etiologies of systemic capillary leak syndrome, underlying malignancy is one of the secondary causes. However, small cell lung cancer is not a malignancy described in either systematic review [3,6]. In addition, the other drugs, including the chemotherapy agents, are not typically identified as causes of capillary leak syndrome [3]. Patients with capillary leak syndrome can also have associated monoclonal gammopathy, which was not addressed in our patient [6].

Regarding the adverse effect of leukocytosis becoming greater than 100,000 cells/µL, there are few case reports discussing the occurrence of this with G-CSF agents [9,10]. Leukocytosis can further complicate the diagnosis of systemic capillary leak syndrome because of the overlap of symptoms with septic shock. This requires a review of the patient’s history and medications while also ruling out infectious etiologies. In our case, our patient’s leukocytosis improved with fluids and other supportive care.

The concurrence of both systemic capillary leak syndrome and leukocytosis greater than 100,000 cells/µL in the setting of pegfilgrastim use has not been previously described in the literature. Given the mechanism of action for pegfilgrastim, the possibility of developing leukocytosis is consistent with its purpose to proliferate neutrophils. The association of pegfilgrastim causing capillary leak syndrome is less understood, but it is theorized that systemic capillary leak syndrome is caused by increased cytokine production, causing vascular permeability [11]. With the association with leukocytosis, this could potentially point to capillary leak syndrome as an inflammatory process, as suggested by one systematic review [3]. In addition, the simultaneous presentation of systemic capillary leak syndrome and leukocytosis could affect the course of treatment because of the similar symptoms to septic shock and thus mask the true underlying etiology. Part of the additional interventions for systemic capillary leak syndrome is steroids and intravenous immunoglobulins (IVIG) [3]. Our patient was appropriately treated with fluid resuscitation and vasopressor support for shock, which overlaps with general treatment for systemic capillary leak syndrome. Furthermore, our patient was treated for COPD exacerbation with methylprednisolone, which incidentally serves as one of the supportive treatments for capillary leak syndrome [3]. This could explain why our patient did not develop the severe complications associated with the recovery phase. In addition, our patient did not develop an episode of recurrent capillary leak syndrome. While steroids could also induce leukocytosis, they appeared to improve our patient’s leukocytosis along with fluid resuscitation. Our patient’s symptoms for systemic capillary leak syndrome and leukocytosis incidentally resolved as part of this management. However, it is important to recognize systemic capillary leak syndrome early due to early intensive therapy being associated with an improved mortality rate of approximately 70% as well as the possibility of the actual mortality rate being higher since this is an underrecognized condition [11]. Therefore, early recognition for systemic capillary leak syndrome is important because of how it affects the course of treatment with the possibility of needing to initiate steroids or IVIG in addition to fluid resuscitation and vasopressor support.

## Conclusions

Pegfilgrastim is an important agent used in multiple settings, including chronic neutropenia, during chemotherapy induction, post bone marrow transplant, and various other conditions. Systemic capillary leak syndrome is underrecognized and a diagnostic challenge for patients who present with shock-like symptoms, especially with underlying comorbidities of heart failure and chronic obstructive pulmonary disease. This can further be complicated by the onset of leukocytosis, which could falsely suggest a new-onset infection. Our patient was treated with supportive care, including norepinephrine, fluids, and steroids, which overlap with the management of systemic capillary leak syndrome. Clinicians should be aware that pegfilgrastim can cause both of these adverse effects and that an appropriate medical history is paramount for early intervention to prevent mortality.
